# Understanding the molecular basis of autism in a dish using hiPSCs-derived neurons from ASD patients

**DOI:** 10.1186/s13041-015-0146-6

**Published:** 2015-09-30

**Authors:** Chae-Seok Lim, Jung-eun Yang, You-Kyung Lee, Kyungmin Lee, Jin-A Lee, Bong-Kiun Kaang

**Affiliations:** Department of Biological Sciences, College of Natural Sciences, Seoul National University, Gwanangno 599, Seoul, Gwanak-gu 151-747 Korea; Department of Biological Sciences and Biotechnology, College of Life Science and NanoTechnology, Hannam University, Jeonmin-dong 461-6, Daejeon, Yuseong-gu 305-811 Korea; Department of Anatomy, Kyungpook National University Graduate School of Medicine, Dongin-dong 2-101, Daegu, Jung-gu 700-422 Korea

**Keywords:** Autism spectrum disorder (ASD), Cellular reprogramming, Induced pluripotent stem cells (iPSCs), Neural differentiation

## Abstract

Autism spectrum disorder (ASD) is a complex neurodevelopmental disorder characterized by deficits in social cognition, language development, and repetitive/restricted behaviors. Due to the complexity and heterogeneity of ASD and lack of a proper human cellular model system, the pathophysiological mechanism of ASD during the developmental process is largely unknown. However, recent progress in induced pluripotent stem cell (iPSC) technology as well as *in vitro* neural differentiation techniques have allowed us to functionally characterize neurons and analyze cortical development during neural differentiation. These technical advances will increase our understanding of the pathogenic mechanisms of heterogeneous ASD and help identify molecular biomarkers for patient stratification as well as personalized medicine. In this review, we summarize our current knowledge of iPSC generation, differentiation of specific neuronal subtypes from iPSCs, and phenotypic characterizations of human ASD patient-derived iPSC models. Finally, we discuss the current limitations of iPSC technology and future directions of ASD pathophysiology studies using iPSCs.

## Introduction

Autism spectrum disorder (ASD), which is characterized, in varying degrees, by difficulties in social interactions, verbal and nonverbal communications, and by repetitive behaviors, is complex disorders of brain development. The prevalence of ASD is estimated to range between ~25 and ~110 per 10,000 children [1, 2]. There are no available cures for this devastating disease despite several current clinical trials. ASD is known to be highly heritable, as indicated by a study of monozygotic twins with a 70–90 % concordance rate. In addition to its strong heritability, recent genetic studies have shown that ASD has hundreds of candidate genes with many different putatively disruptive variants [3, 4]. However, these are relatively rare genetic variations, each of which accounts for less than 1 % of ASD cases [5]. Furthermore, ASD-associated genetic variations occur *de novo* in affected individuals and are sometimes inherited from normal parents, indicating either incomplete penetrance or other genetic modifications. Current studies have focused on the identification of common cellular pathways in order to account for connections between these various ASD candidate genes. Interestingly, to date, many synaptic proteins have been identified as ASD candidate genes, making it possible to study ASD pathogenesis using cellular and animal models [6–9].

To understand the underlying pathophysiological mechanisms of ASD, murine models have been generated using ASD candidate genes, including synaptic genes [10–12]. However, murine models are not always feasible and have several limitations for studying human neurodevelopment. Heterozygous mice with ASD mutation rarely develop ASD phenotypes unless the ASD genetic mutation is homozygous, which is exceptionally rare in ASD cases, indicating that other genetic modifications are required for developing ASD phenotypes or candidate genes have different functions in human neurons [13]. Furthermore, some human neocortical regions affected in ASD are not obtainable from mouse brain tissue, and brain development of mice does not perfectly reflect typical development of the human brain. Thus, understanding of neurodevelopmental disorders such as ASD has been lagged in the studies using animal models, including rodents or primate [14, 15]. Although primate models can overcome the limitations of rodent models such as differences in brain anatomy, response to drugs, or circuit connectivity between human and rodent brains, they recapitulate only limited behaviors such as simple social interactions or repetitive behaviors. Primate models could be difficult to apply for representation of a variety of human complex behavioral alterations shown in ASD patients to understand associated biological mechanisms and develop a knowledge-based therapy for ASD [15]. Although *in vitro* studies on neural differentiation using human embryonic stem cells (ESCs) have been suggested for understanding of human neurodevelopment, there remain numerous practical or ethical issues [16, 17].

To overcome these obstacles, induced pluripotent stem cells (iPSCs) technology, which allows the generation of personalized human neurons from ASD patients, has been used for studying the pathophysiology of ASD [18–20]. In this case, human neurodevelopment, which cannot be addressed in an animal model *in vitro* or *in vivo*, can be tracked using personalized iPSCs from ASD patients under an individual genetic background. Moreover, current gene engineering technology for human iPSCs using sequence-specific designed zinc finger nuclease (ZFN), transcription activator-like effector nuclease (TALENs), or CRISPR/Cas-9 has made disruption, mutation, or deletion of even large genomic fragments possible at a specific locus in the genome of hiPSCs and can be applied in ASD research for generation of isogenic iPSCs with gene correction and genetic disruption [21–26]. In addition, as an alternative method for customized disease modeling, direct conversion methods from human somatic cells into desired cell types such as neurons using lineage-specific factors have been suggested [27–29], although this method is still challenging and further stabilizing steps are needed for standardization of protocols.

In this review, we summarize (1) recent advances in generation of iPSCs, (2) current methods of neural differentiation from iPSCs, and (3) functional characterization of cellular disease phenotypes using recent ASD iPSC models and then discuss current limitations, future directions for modeling of ASD using iPSC technology, and potential applications [26, 30].

## Generation of iPSCs from human somatic cells: cellular reprogramming

**Fig. 1:**
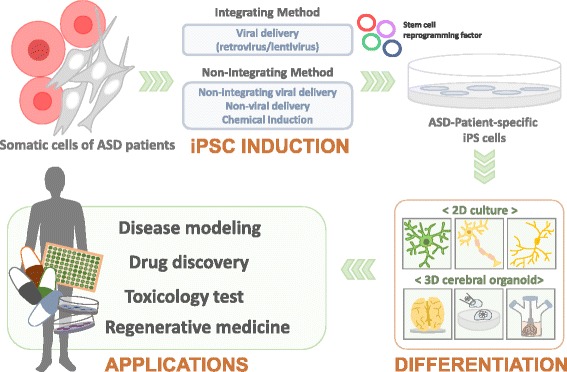
Generation and application of patient-specific iPSCs. Patient-specific iPSCs have been generated from human somatic cells such as skin fibroblasts or peripheral blood cells by viral, non-viral delivery, or chemical induction method. These customized iPSCs have been differentiated into desired neuronal cell types such as neurons, astrocytes, or microglia. Furthermore, iPSC-derived EB (embryoid body) could develop 3D cerebral organoids, which can recapitulate human cortical development. Therefore, patient-specific iPSC-derived neural cells or cerebral organoids could be used for diverse applications for disease modeling, drug discovery, toxicology test, and regenerative medicine

In 2006–2007, Takahashi and Yamanaka first showed that retroviral transfer of four transcription factors (Oct4, Sox2, Klf4, and c-Myc) known as Yamanaka’s factors is sufficient for cellular reprogramming of mouse or human skin fibroblasts into stem-cell like cells known as iPSCs, which have self-renewability and pluripotency [31, 32]. Although there are concerns about subtle differences in transcriptomes, proteomes, and epigenomes between ESCs and iPSCs, iPSCs have been used in diverse research areas and clinical trials such as disease modeling, drug discovery, toxicology test, and regenerative medicine [26, 33] (Fig. 1). In recent years, iPSC reprogramming technology has undergone considerable improvements to overcome inefficient protocols and ensure functional derivatives for clinical application. Recent developments in iPSC technology using various somatic cell types include improved reprogramming methods using novel delivery systems such as non-integrating viral and non-viral vectors as well as identification of alternative reprogramming factors or small molecules such as inhibitors of specific signaling or epigenetic modulators, which replace conventional reprogramming factors and facilitate reprogramming processes [33–35] (Table 2). A number of studies have reported detailed protocols for iPSC generation [35, 36]. Here, we summarize recent trends for generation of iPSCs from human somatic cells.

### Sources of somatic cells for reprogramming

The first step in iPSC generation is obtaining appropriate human somatic cells from patient tissues [37, 38] after an accurate diagnosis of disease based on valid clinical processes. However, unlike other genetic or non-psychiatric disorders, the examiners should be far more careful when diagnosing ASD, including autism. Clinicians can usually consider two different categories of behavioral tests for the diagnosis of autistic individuals, observational reports (including questionnaires) such as the Autism Diagnostic Observation Schedule (ADOS) [39], Autism Diagnostic Interview-Revised (ADI-R) [40], Clinical Global Impression (CGI) Scale [41], Childhood Autism Rating Scale (CARS) [42, 43], Autism Behavior Checklist (ABC) [44, 45] and Social Responsiveness Scale (SRS) [46, 47] and the results of an aptitude test such as the Wechsler Adult Intelligence Scale (WAIS) [48] (Table 1).

**Table 1:** Behavioral tests for the diagnosis of autistic individuals

	Test methods	Description	References
Observational report (including questionnaires)	Autism Diagnostic Observation Schedule (ADOS)	A standardized assessment in terms of objective evaluation of autistic social and communicative behavior symptoms	Lord et al. 1989 [39]
	Autism Diagnostic Interview-Revised (ADI-R)	An interview conducted with the parents of autistic individual to cover autistic individual’s full developmental history	Lord et al. 1994 [40]
	Clinical Global Impression (CGI) Scale	A three-item scale used to assess treatment response in patients with mental disorders	Guy 1976 [41]
	Childhood Autism Rating Scale (CARS)	A score calculated by subjective observation of a child’s behavior across fifteen criteria	Schopler et al. 1980 [42]; Mayes et al. 2012 [43]
	Autism Behavior Checklist (ABC)	A 57-checklist of behavioral characteristics, which has been used for diagnosis of autism	Krug et al. 1980 [44]; Volkmar et al. 1988 [45]
	Social Responsiveness Scale (SRS)	A quantitative measure of autistic traits completed by a caregiver familiar with the autistic individuals within 4–18 year olds	Constantino 2002 [46]; Aldridge et al. 2012 [47]
Aptitude test	Wechsler Adult Intelligence Scale (WAIS)	A form of IQ test designed to measure intelligence in adults and older adolescents, which has separate verbal and non-verbal IQ scores	Wechsler 1939 [48]

The most common cell type as the starting material for reprogramming process is skin dermal fibroblasts [38]. However, since it is difficult to obtain skin biopsies from young children, especially those with autistic behavior, and the skin biopsy method using a punch is an invasive approach, it is important to obtain donor cells with high reprogramming capacity in a non-invasive way. As an alternative common cell source, peripheral blood cells are used for cellular reprogramming due to their non-invasive, easy, and routine accessibility in a clinic setting [38, 49, 50]. Recent efforts in iPSC generation have attempted to develop exfoliated renal epithelial cells from urine, buccal cells, cord blood-derived cells, or keratinocytes from hair cells as cell sources for reprogramming despite concerns about purification method, culture stability for long-term passaging, reproducibility, and efficiency for reprogramming [38]. Future advances in iPSC technology using human somatic cell types with easier access and handling, higher efficiency, and cost-effectiveness for successful reprogramming will allow development of more common customized medicines using iPSCs.

### Induction methods for cellular reprogramming: integrative/non-integrative

Once somatic cells are taken from biopsies and cultured enough passages, they can be induced into stem cells using an appropriate reprogramming method. Many kinds of induction methods for cellular reprogramming have been reported depending on the delivery system of reprogramming factors or types of factors (ex, small molecules, inhibitors, etc.) as alternative reprogramming inducers [33]. The most common method is the introduction of reprogramming factors into somatic cells via an integrating or non-integrating delivery system [33, 51, 52].

#### Integrating delivery system: retroviral/lentiviral vectors

Integrating methods use viral vectors such as retroviral or lentiviral vectors with high efficiency of gene delivery, although viral vectors integrate into the host cell genome (reprogramming efficiency: ~0.01–0.1 %). Generally, reprogramming factors are silenced after cellular reprogramming. However, genomic integration using viral vectors could induce reactivation of these genes, raising the possibility of oncogenesis in iPSC–derived cells or silencing of other functional genes after cellular reprogramming [53, 54]. Thus, many reprogramming methods without genomic integration have been described as a non-integrating approach, and some are commonly used for generation of iPSCs [26, 33, 36, 52].

#### Non-integrating delivery system

Regarding non-integrating approaches, non-integrating viral vectors (Sendai or Adeno virus), episomal vector, piggy BAC vector, Cre-inducible/excisable lentiviral vector, minicircle DNA, poly-arginine-tagged polypeptide (protein), RNA-modified synthetic mRNA, or microRNAs have been suggested for iPSC generation with diverse ranges of reprogramming efficiency (reprogramming efficiency: 0.001 ~ 4 %) [33, 35, 55–65]. Although each method has pros and cons (Table 2), non-integrating approaches generally have lower efficiency of cellular reprogramming compared to integrating lenti- or retroviral vectors. However, many efforts have attempted to improve the efficiency of cellular reprogramming.

**Table 2:** Comparisons of reprogramming delivery system

	Delivery system	Pros	Cons	References
Integrating method	Retrovirus	High reprogramming efficiency (~0.01–0.1 %)	Possibility of oncogenesis; silencing of functional genes	Takahashi and Yamanaka. 2006 [32]
	Lentivirus	High reprogramming efficiency (~0.01–0.1 %)	Possibility of oncogenesis; silencing of functional genes	Yu et al. 2007 [51]
Non-integrating method	Sendai virus	No risk of altering the host genome; high reprogramming efficiency(~1 %); easy to select iPSCs	Stringent steps to remove the reprogrammed cells of replicating virus; sensitivity of the viral RNA replicase	Fusaki et al. 2009 [55]
	Adenovirus	Transient, high-level expression	Low reprogramming efficiency (0.0001-0.001 %); possibility of small pieces insertion of adenoviral DNA; 3 out of 13(or approximately 23 %) were tetraploid	Stadtfeld et al. 2008 [56]
	OriP/EBNA-based episomal vector	Unnecessary for viral packaging; gradual loss of cellular EV without drug selection; relatively high reprogramming efficiency of IRES2-mediated expression(~0.1 %); further addition of c-Myc and Klf4 improve the reprogramming efficiency to over 1 %	Unstable transfection efficiency	Yu et al. 2009 [168]
	Piggy BAC transposons	Technical simplification (use of effortless plasmid DNA preparation and commercial transfection products); no limited range of somatic cell types for reprogramming; allow the option of xeno-free hiPSC production; accurate transgene removal through transposase expression	Labor intensive removal of multiple transposons; more CNVs in early passage than in intermediate passage;	Woltjen et al. 2009 [59]; Hussein et al. 2011 [162]
	Cre-inducible/excisable lentivirus	Minimize the risk of chromosomal translocations; improve the developmental potential and differentiation capacity	Inefficient delivery of Cre; difficult to detect successful Cre-recombeniation; result in mosaic colonies; leaves 200 bp of exogenous DNA	Sommer et al. 2010 [58]; Soldner et al. 2009 [169]; Papapetrou et al. 2011 [170]
	Minicircle DNA	Free of foreign or chemical elements; requiring only a single vector without the need for subsequent drug selection, vector excision, or the inclusion of oncogenes; FAD approved	Low reprogramming efficiency (~0.005 %)	Jia et al. 2010 [73]; Narsinh et al. 2011 [75]
	Poly-arginine-tagged polypeptide	No risk of altering the host genome; simpler and faster approach than the genetic method	Low reprogramming efficiency (~0.006 %); requires either chemical treatment or greater than four rounds of treatment; expertise in protein chemistry and handling	Zhou et al. 2009 [171]; Kim et al. 2009 [60]
	RNA-modified synthetic mRNA	Avoid the endogenous antiviral cell defense; high efficiency of over 2 %; resultant iPSC colonies emerge as early as 17 days	Labor intensive repeated transfection	Warren et al. 2010 [61]
		Non-immunogenic; cost-effective; easily handled;	Relatively low and inconsistent efficiency	Hou et al. 2013 [80]

1. Non-integrating transgene systems: Sendaiviral/Adenoviral vector, episomal vector, integrative but excisable system (piggy Bac, Cre-loxP), and minicircle DNA
  As one of the most attractive non-integrating viral vectors, Sendai virus with a negative-sense single-stranded RNA has been suggested as a potential clinical candidate since replication of transgenes occurs in the cytoplasm without possible genomic integration [55, 66, 67]. Although adenoviral vectors for cellular reprogramming have also been suggested as a non-integrating delivery system due to their transient and high expression of transgenes, reprogramming efficiency of human somatic cells is too low for common use (~0.0002 %) [68]. For transient expression of reprogramming factors, compared to previous episomal vectors, more advanced OriP/EBNA-based episomal vectors delivering combinational transgenes such as OCT3/4, SOX2, KLF4, L-MYC, LIN28, and shRNA for p53 have been described as a promising non-integrating approach for successful iPSC generation with acceptable reprogramming efficiency [57, 69]. Generation of integration-free iPSCs using either piggy Bac transposon or the Cre-loxP system has been also successful. Both systems are known to remove integrating transgenes from iPSCs after reprogramming, although there is a small risk of gene breaks near the insertion site [58, 59, 70, 71]. Recently, minicircle DNA, which is a novel compact vector free of bacterial DNAs or human artificial chromosomes (HACs) with capacity for large gene insertion and stable episomal maintenance, have been used to successfully generate iPSCs, although their low reprogramming efficiency should be improved [72–75].
2. Non-integrating transgene-free systems: modified mRNA, protein, and chemicals
  As for other transgene-free systems, modified mRNA, microRNA, or protein has been suggested as an attractive method for iPSC reprogramming in a clinical application due to more direct delivery of reprogramming factors without genomic integration. Synthetic mRNAs modified to avoid the endogenous antiviral cell defense system have more efficiently generated iPSCs with higher efficiency and faster iPSC induction compared to the retroviral system. However, labor-intensive steps such as repeated transfections of mRNAs should be improved. Reprogramming using microRNAs has also been successful with higher efficiency [62]. The protein transduction method using cell penetrating peptides is one of the safest methods for generating foot-print free iPSCs for use in a clinical purpose although reprogramming efficiency is very low (~0.0001 %) [60]. In this system, technical challenges include generation of a large amount of functionally active and stable proteins as well as induction of reprogramming from diverse types of somatic cell sources via penetrating reprogramming proteins with simple treatment [76].
  Alternatively, diverse chemical compounds capable of replacing initial Yamanaka’s factors or other reprogramming factors have been investigated for iPSC generation due to their non-immunogenic, cost-effective, ease of use, reversible, cell-permeable, and standardized properties despite their inconsistent and low reprogramming efficiency. Small molecules that target signaling pathways such as transforming growth factor β (TGFβ) or epigenetic factors such as histone deacetylase have been proposed to generate iPSCs and improve reprogramming efficiency [76–79]. More recently, a cocktail of chemical compounds without any genetic factors successfully induced iPSCs from mouse somatic cells, raising the possibility of its application in the generation of iPSCs from human somatic cells [80]. More intensive screening for small molecules for cellular reprogramming and optimization is needed for efficient iPSC generation and its suitable application.
  As mentioned above, a variety of promising methods with advantages and disadvantages have been proposed for the generation of patient-specific iPSCs (Table 2). Recent systematic evaluation of the most widely used techniques (Sendai-viral, episomal, or transfection of mRNA methods) for generating transgene-free hiPSCs have shown that significant differences between methods include aneuploidy rates, reprogramming efficiency, reliability, and workload, although they all result in high-quality iPSCs [81]. iPSC technology is rapidly advancing toward a transgene-free, small-molecule-based approach using diverse types of human somatic cells. Choice of reprogramming method will depend on the specific purposes for one’s own iPSC research. For basic research or drug/toxicology tests using iPSCs, reprogramming methods generating iPSCs such as cost-effective integrating/nonintergrating methods with higher efficiency could be selected based on reprogramming efficiency, workload, time or economic feasibility, regardless of its safety issues. However, for clinical applications using iPSCs, safety issues such as caner progression, purity, or accessibility and feasibility using patient samples would be the most important concerns influencing selection of reprogramming methods, which would be nonintegrating/transgene-free methods.

## Generation of iPSC-derived neurons: neural differentiation

In disease modeling using patient-specific iPSCs, the most important step is to differentiate iPSCs into desired cell types with high purity. Accumulating research on vertebrate neural development has enabled us to generate specific subtypes of human neurons or glial cells from human pluripotent stem cells (PSCs) by regulating developmentally relevant signaling pathways. During embryonic development, the neural plate (embryonic neuroectoderm) is firstly specified to the forebrain, subsequently to the midbrain/hindbrain, and then to the spinal cord by caudalization signals that include retinoic acid (RA). Similarly, human PSCs can be directed to differentiate into forebrain-like neurons by inhibiting Wingless/Int proteins (Wnt) and bone morphogenic protein (BMP) signaling [82], midbrain/hindbrain by sonic hedgehog (SHH) and fibroblast growth factor 8 (FGF8) treatment [83, 84], and spinal cord by the action of RA *in vitro* [85, 86].

### Two-dimensional neural differentiation

**Fig. 2:**
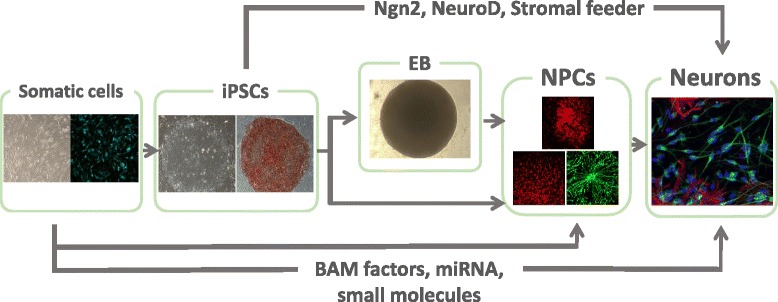
Neural differentiation from iPSCs. To study the pathophysiology of ASD using iPSCs-derived neurons, iPSCs need to be differentiated into the disease-relevant neuronal subtype such as cortical neurons. There are general methods currently used for neural induction through embryoid body (EB) formation, cultivation on stromal feeder cells, direct differentiation of iPSCs into neural lineage by lineage specific factors such as *Ngn2* or *NeuroD1*, or direct conversion of somatic cells into neurons by expression of BAM factors and/or microRNAs

For disease modeling using iPSC-derived neurons, specific subtypes of neurons differentiated from iPSCs should be carefully chosen since the affected cell types and brain areas are different. A variety of subtype-specific neural differentiation protocols have been developed based on embryonic developmental studies. There are three general methods currently used for neural induction: (i) through embryoid body (EB) formation [85–90], (ii) cultivation on stromal (or mesenchymal) feeder cells [83, 91, 92], and (iii) direct conversion into neural lineage by lineage-specific factors [93–97] or small molecules [98–100] (Fig. 2).

#### EB-dependent differentiation

*In vivo* neural tissue is differentiated from a germ layer called the ectoderm. Similarly, stem cells *in vitro* can develop three germ layers within aggregates called EBs, including ectoderm under appropriate stimulating conditions. Neural induction of stem cells can be commenced via EB formation by low basic fibroblast growth factor (bFGF) and subsequent induction of EBs into neural rosettes, which are a polarized organization of neuroepithelial cells and neural differentiation is achieved by a combination of specific sets of morphogens such as Wnt, SHH, BMPs, RA, and FGFs [85–88, 101–103]. Motor neurons having spinal lateral column phenotypes and midbrain dopaminergic neurons were differentiated from hESCs or hiPSCs with the EB formation method [84, 101, 102]. Most *in vitro* ASD-related disease modeling has been used this method (Table 3). For example, GABA (γ-amino butyric acid) and vGlut1 (vesicular glutamate transpoter-1)-positive neurons were differentiated through EB formation [104, 105] and by blocking the BMP pathway [106] in *in vitro* Rett syndrome modeling. In addition, Dolmetsch group used this method to obtain vGlut1/2-, GAD65/67-positive and lower cortical layer-enriched neurons from syndrome patient-derived iPSC for investigation of Timothy syndrome [107, 108]. Tuj1-, MAP2- or GFAP-positive cells from Fragile-X syndrome patient-derived iPSCs [109, 110] and Tuj1-positive cells from Angelman syndrome patient-derived iPSCs [111] were also differentiated with this method. *In vitro* neuronal induction through EB formation, however, is time-consuming and requires multiple steps for generation of neural epithelial cells, neuronal progenitors, neuronal differentiation, and synaptic connection and maturation. Furthermore, it is hard to obtain a homogenous population of desired neuronal subtypes with high purity due to the difficulty in controlling specific lineage differentiation from EBs [112]. Therefore, the general neural induction method from EB formation was recently modified to improve induction efficiency and purity of desired neuronal cell types as well as reduce multiple steps for neural induction, although the neural induction method through EBs *in vitro* is ideal to mimic *in vivo* neural induction and neuronal differentiation. For example, inhibition of TGFβ and BMP pathways (dual SMAD inhibition: noggin and SB431542) have been used for efficient neural induction from stem cells without an EB formation step [113–116]. For in vitro modeling of Phelan-Mcdermid syndrome, Dolmetsch group used this method, with some modifications, to generate cortical neurons [117] (Table 3).

**Table 3:** Phenotypic analyses of ASD iPSC-derived neurons : Rett, Phelan-Mcdermid, Timothy, Fragile-X, and Angelman Syndromes

Diseases	Related genes	Neural differentiation methods	Identity of neurons	Electrophysiological proterties	Neurodevelopmental phenotypes	References
Rett syndrome	Methyl CpG binding protein 2 (MECP2)	Embryoid body formation	Gluramatergic & gabaergic neurons	Reduced sEPSC and sIPSC	Fewer synaptic conracts; reduced cell soma size and dendritic branching and spine density	Marchetto et al. 2010 [104]; Cheung et al. 2011 [105]; Kim et al. 2011 [106]
Phelan-McDermid Syndrome (PMDS) (22q13 deletion syndrome)	Shank3	Dual smad inhibition	Forebrain neurons	Reduced excitatory synaptic transmission	Reduced glutamatergic receptors; decreased number of synapses	Shcheglovitov et al. 2013 [117]
Timothy syndrome (A member of the long QT syndromes)	CACNA1 (alpha-1 subunit of the L-type calcium channel CaV1.2)	Embryoid body formation	Cortical-enriched neuronal populations	Increase in the sustained intracellular calcium rise following membrane depolarization; wider action potentials	Decreased expression of lower corticallayers-related genes; increases in TH (tyrosine hydroxylase)-, norepinephrine- and dopamine-positice cells; activity-dependent dendrite retraction	Pasca et al. 2011 [107]; Krey et al. 2013 [108]
Fragile X syndrome	Fragile X mental retardation 1 (FMR1)	Embryoid body formation	Tuj1-, MAP2- or GFAP-positive cells	Poor spontaneous synaptic activity and no glutamate reactivity	Reduced neurite numbers and neurite lengths; reduced PSD95 protein expression and reduced synaptic punctadensity; poor neuronal maturation and high gliogenic development	Sheridan et al. 2011 [109]; Telias et al. 2013 [110]
Angelman syndrome	Ubiquitin protein ligase E3A (UBE3A)	Embryoid body formation	Tuj1-positive cells	Normal electrophysiological properties	Intact imprinting of UBE3A	Chamberlain et al. 2010 [111]

#### Cultivation on stromal (or mesenchymal) feeder cells

As the other neural induction method, stromal feeder-based differentiation system, which is a serum-free system without the use of either RA or EBs, has been widely used, although the molecular basis of the neural-inducing activity of stromal cells remains unclear [83, 92]. An initial study suggested that stromal cells induce midbrain neuronal fate by default [91].

#### Direct conversion: somatic or pluripotent stem cells to neurons/somatic cells to neural progenitor or neural stem cells

The other approach to generate human neurons is to convert human stem cells or somatic cells directly into neurons by defined specific factors [93–96] or small molecules [98–100]. Wernig’s group reported a simpler and direct neural conversion method from human PSCs by forced expression of only a single transcription factor, *Neurogenin 2* (*Ngn2*), *NeuroD1* [97] or *ASCL1* [118]. In human neurons induced via this method, functional synapses are rapidly formed within only 2 weeks after neural induction so that the time required to obtain mature human neurons *in vitro* is significantly reduced. Moreover, the most attractive point of using this method is to obtain a homogeneous cell population (~100 % of cortical neurons) differentiated from hESCs and hiPSCs [97]. As an alternative approach to generate induced neurons, a combination of three transcription factors - *BRN2* (also called *Pou3f2*), *ASCL1* (also known as *MASH1*), and *MYT1L* (so called BAM factors) - could convert adult mouse fibroblasts directly into functional neurons without iPSC generation [93, 119]. The neurons generated by this method are also able to fire spontaneous action potentials and make functional synapses within as early as 2 weeks after induction *in vitro*. The same three transcription factors also could differentiate human stem cells and fibroblasts into neurons when combined with a transcription factor, *NeuroD1* [94], microRNAs [95, 120], or small molecules [121]. In addition, very recently, it has been reported that only small-molecule cocktails were sufficient to directly convert mouse and human fibroblasts to functional neurons without exogenous genetic factors [98–100]. Likewise, rapid generation of specific subtypes of neurons directly from somatic cells makes this method an effective strategy for *in vitro* ASD modeling. However, a key limitation of this method is that a large number of fibroblasts might be required for reliable experiments due to their low reprogramming efficiency (at most 10–30 %), and skin biopsy cannot be conducted many times on a single patient. Therefore, in some cases, it would be desirable to convert fibroblasts into self-renewing multipotent neural progenitor cells (NPCs) or neural stem cells (NSCs), which enables us to overcome the limitations associated with low reprogramming efficiency and thereby perform high-throughput drug screening. Kim et al. [122] described the generation of NPCs from mouse fibroblasts by transient expression of Yamanaka’s factors (Oct4, Sox2, Klf4, and c-Myc), followed by culturing in neural induction media. However, NPCs generated by this method could be expanded for only a few passages. Thier et al. [123] have generated induced NSCs with the same classical factors (Oct4, Sox2, Klf4 and c-Myc) by strictly limiting Oct4 expression and optimizing culture conditions. In addition, forced expression of four transcription factors (BRN4/Pou3f4, SOX2, KLF4 and c-MYC) [124] or even a single transcription factor SOX2 [125] could also directly convert mouse or human fibroblasts into NSCs without generating a pluripotent cell state. Therefore, this direct conversion method is considered a promising method for preventing teratoma formation, which is a disadvantage of iPSCs for regenerative medicine, as well as for greatly improving low conversion efficiency from fibroblasts to neurons [126].

To study the pathophysiology of ASD using iPSC-derived neurons *in vitro*, it is important to obtain desired homogeneous neurons associated with ASD, as mentioned above. Cortical neurons have been suggested to be appropriate cell types since potential mechanisms underlying ASD include defects in cortical connectivity and neural migration to the cerebral cortex [127]. Moreover, despite the heterogeneity of ASD, common pathways involved in synaptic development and plasticity have been proposed to be deregulated in ASD. Thus, to study developmental synaptopathy in ASD, among several protocols for neural induction, rapid generation of human cortical neurons using defined factors could be one of the best strategies for *in vitro* ASD modeling due to their high induction efficiency of homogenous neuronal subtype and short induction time. However, if human neurons are directly generated from stem cells or somatic cells for modeling neurodevelopmental disorders such as ASD, it might be difficult to detect developmental phenotypes during neural differentiation. Furthermore, continuous forced expression of defined factors could also mask disease phenotypes [20, 128]. Therefore, differentiation efficiency or stability of human neurons induced by defined factors should be improved, and comparable systematic analysis of neuronal properties such as gene expression, electrical properties, or synaptic connections in human neurons differentiated either through EB formation or by defined factors needs to be carried out.

### Three-dimensional neural differentiation: cerebral organoids

Magnetic resonance imaging (MRI) studies and postmortem analysis of individual patients with ASD have consistently demonstrated anatomical abnormalities in several brain regions, which cannot be recapitulated by two-dimensional (2D) iPSC-derived neuronal culture [129]. An iPSC-derived three-dimensional (3D) culture system termed cerebral organoid has been developed [130, 131]. Cerebral organoids, which develop through intrinsic self-organizing properties, can be generated from EBs grown initially in ESC medium with low bFGF and Rho kinase (ROCK) inhibitor [131], and they have been shown to recapitulate the complex interplay of different regions and structures of the brain [130]. Therefore, 3D cerebral organoids derived from ASD patient-specific iPSCs would be the best *in vitro* model to uncover defects in cortical connectivity and neuronal migration of ASD. Indeed, Mariani et al. recently generated idiopathic ASD patient’s iPSC-derived brain organoids and showed increased production of inhibitory neurons by increased FOXG1 gene expression [132]. However, more standardized protocols need to be developed, and further characterization and identification of neuronal cell types in specific regions of cerebral organoids should be carried out to study cortical development and for disease modeling of ASD patients.

## Analyses of human iPSC-derived neurons

To use iPSC technology in modeling of various neurodevelopmental disorders including ASD *in vitro*, it is important to characterize disease phenotypes in disease-specific iPSC-derived neurons and validate well-known disease phenotypes to determine whether or not iPSC-derived cellular disease models could recapitulate disease phenotypes in mouse models and human patients. Because of this reason, ASD research using this technology primarily includes several studies on monogenic cases, such as Rett Syndrome, Fragile X Syndrome, and Timothy Syndrome [104–108, 133, 134]. However, these initial studies on cellular disease phenotypes in iPSC-derived neurons from monogenic cases of ASD could be directed towards the identification of disease-relevant cellular characterization in both monogenic and idiopathic forms of ASD with high heterogeneity. In this section, we describe what phenotypic analyses of human iPSC-derived neurons can be performed to characterize and validate iPSC-derived cellular disease models.

**Fig. 3:**
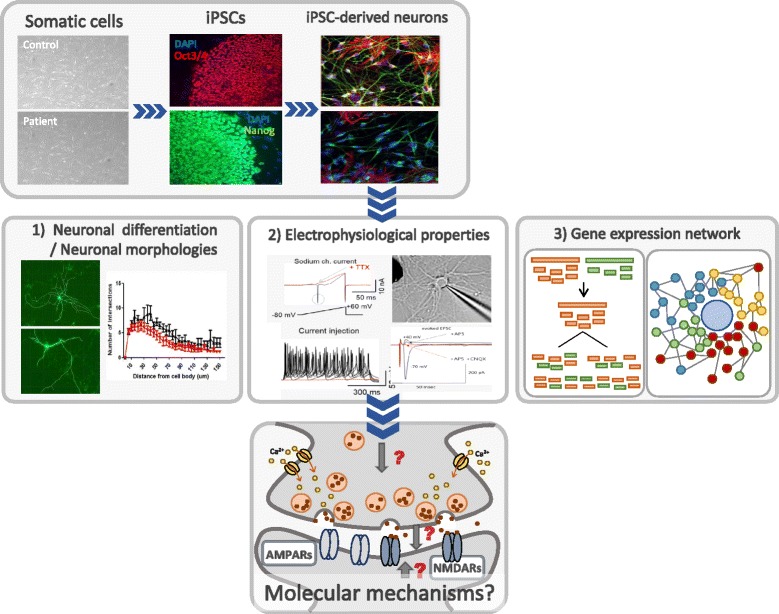
Phenotypic analyses of iPSC-derived neurons. Patient-specific iPSCs are generated from human somatic cells. After characterization, expansion, and stabilization of iPSCs, ASD patient-derived human neurons are induced. These differentiated neurons can be characterized by changes in neuronal differentiation, morphological properties, electrophysiological properties, or gene expression network to elucidate molecular pathogenic mechanisms associated with ASD such as synaptopathy

There are general phenotypic analyses of human neurons derived from iPSCs based on (i) neural differentiation and neuronal morphologies (neurite outgrowth, synapse structure), (ii) electrophysiological properties (basic electrical properties, synaptic properties), and (iii) gene expression network (transcriptome analysis) (Fig. 3).

### Neural differentiation and neuronal morphologies: neurite outgrowth/synapse structure

Fully differentiated neuronal cells have a distinct morphology, including distinct polarity, and extend one axon and dendritic arbors from their cell body. Therefore, the earliest phenotypes of hiPSC-derived neurons are morphological changes such as neural differentiation, neurite/axon/dendritic growth (number or length of neurite process), and synapse formation, which can be used for analysis of disease-relevant morphological phenotypic changes. During the early stages of neurogenesis, newborn neurons are NeuN-positive [135] and PSA-NCAM-positive [136]. These markers, together with neuronal cytoskeletal proteins Tuj1, Tau, and MAP2, can be used for measuring neural maturation efficiency or morphological changes in ASD iPSC-derived neurons [137]. Specific neuronal gene expression as a subtype-specific marker can be also used to confirm neuronal identities. For example, glutamatergic neurons can express vGlut1 and vGlut2 [138], GABAergic neurons express GAD65/67 [139], and mature dopaminergic neurons express tyrosine hydroxylase (TH) [140].

Moreover, disease phenotypes such as cortical connectivity and neural migration in ASD-derived cerebral organoids would be characterized using various markers for a specific subtype of neurons in cortical regions. In rodents, cortical glutamatergic neurons can be defined by their expression of different transcription factors [17, 141–148]. Layer 6 corticothalamic projection neurons are *Tbr1*-positive [144, 145], layer 5 subcortical projection neurons are *Ctip2*-positive [143], layers 2–4 neurons are *Cux1/2*-positive [146], and layers 2–4 callosal projection neurons are *Satb2*-positive [147, 148].

### Electrophysiological properties

Electrophysiological characterization for basic electrical properties such as membrane potential, generation of action potentials by current injection, and synaptic properties such as appearance of spontaneous synaptic events can be applied to verify and characterize hiPSC-derived neurons. As neurons mature, resting membrane potentials (V_m_) become negative (more hyperpolarized) and capacitance (C_m_) increases due to increased branch numbers, leading to increased cell volume. In contrast, input resistance (R_i_) decreases as channel protein expression increases. In addition, action potential-like responses induced by depolarization are resemble the mature shape of the action potential. Since neurons are able to make synapses with other neurons, synaptic properties could be also characterized as a cellular phenotype in iPSC-derived disease models. hiPSC-derived neurons from many syndromic ASD patients have defects in synaptic connectivity such as spontaneous excitatory and inhibitory currents [104], AMPA/NMDA current ratio [117], as well as intrinsic neuronal excitability [107] (Table 3).

### Gene expression network

Similar to the phenotypic analyses, transcriptional changes based on gene expression network could be characterized in ASD iPSC-derived neurons. This analysis based on a systems biology approach allows us to understand alterations of the gene network involved in neural development and functions associated with ASD. Recent studies using genome-wide weighted co-expression network analysis (GWCNA) on Timothy Syndrome (TS)-derived neural cells have shown that altered Ca^2+^ signaling in TS patients leads to dysregulation of calcium-dependent transcriptional regulators such as NFAT, MEF2, CREB, and FOXO as well as its downstream signals [149]. Gene expression analysis of hiPSC-derived neurons carrying copy number variants of chromosome 15q11-q13.1 using RNA-Seq has revealed that common neuronal pathways may be disrupted in both Angelman and Dup15q syndromes [150].

### Phenotypic analyses of ASD iPSC-derived neurons: Rett, Phelan-Mcdermid, Timothy, Fragile-X, and Angelman Syndromes

In Rett syndrome (RTT), a neurodevelopmental ASD due primarily to mutations in the methyl-CpG binding protein 2 (MECP2) gene, hiPSC-derived neural cells show reduced soma size, dendrite spine density, differentiation, and reduced spontaneous Ca^2+^ transient frequency in neurons and premature astroglial [104–106, 151, 152], as shown in human postmortem analyses. In addition, reduced frequency and amplitude of mEPSCs and mIPSCs [104] have been observed in RTT hiPSC-derived neurons, suggesting fewer production of synapses and reduction of postsynaptic receptors [104]. HiPSC-derived cellular disease models could be also used as a system for screening candidates for disease therapy since iPSC-derived neurons can recapitulate disease phenotypes in human and mouse models. Indeed, insulin-like growth factor 1 (IGF-1) was applied to hiPSC-derived RTT neurons and showed rescue of reduction in excitatory glutamatergic synaptic connections [104] as in mouse models of RTT, in which reduced excitatory synaptic connections in RTT neurons could be reversed by IGF-1 application [153]. IGF-1 is currently in clinical trials for RTT.

hiPSC-derived neurons from Phelan-Mcdermid syndrome (PMDS) patients, carrying a deletion of Shank3 protein, have significant deficits in excitatory synaptic transmission [117]. These deficits were rescued by either wild-type Shank3 expression or IGF-1 treatment as in hiPSC-derived RTT neurons [117], suggesting that a common signaling pathway might be involved in the pathophysiology of ASD. Therefore, hiPSC-derived neurons could be useful as a potential drug-screening platform, as mentioned above.

Timothy syndrome (TS) is caused by a point mutation in the voltage-gated calcium channel encoded by the *CACNA1* gene. TS patients iPSC-derived neurons show wider action potentials, suggesting a loss of CaV_1.2_ channel inactivation, abnormal expression of tyrosine hydroxylase (TH), and increased production of norepinephrine and dopamine [107]. Activity-dependent dendritic retraction by RhoA activation independent of Ca^2+^ influx through CaV_1.2_ has also been reported in TS iPSC-derived neurons [108].

Neurons from Fragile-X syndrome patients-derived hiPSCs show reduced neurite numbers and lengths [109], poor spontaneous synaptic activity, and lack reactivity to glutamate [110].

In Angelman Syndrome (AS), which is caused by reduced expression of the maternal copy of the *Ube3A* gene in CNS, patient-derived iPSCs show retained genomic imprinting. In addition, electrophysiological recordings have detected AMPA receptor-mediated spontaneous activity in AS iPSC-derived mature neurons, suggesting that normal functional neurons can be generated from AS iPSCs [111].

Recently, Muotri’s group have generated an iPSC model of a nonsyndromic ASD patient carrying a *de novo* balanced translocation transient receptor potential channel 6 (TRPC6) [154]. TRPC6-mutant iPSC-derived neurons showed reduced neuronal arborization, fewer dendritic spines and synapses, and impaired calcium dynamics [154]. They also found that MECP2 occupied the *TRPC6* promoter region and regulated TRPC6 expression, raising the possibility of interactions among common pathways affected in nonsyndromic and syndromic ASD.

## Conclusions

### Perspectives: limitations and future directions

#### iPSC research

Despite numerous studies underlying the pathophysiological mechanism of ASD using iPSCs, several concerns should be addressed before iPSC research [155, 156]. Current advances in iPSC technology have allowed us to successfully derive patient-specific iPSCs regardless of their reprogramming methods. Furthermore, a recent study showed that a modular, robotic platform for iPSC reprogramming enabled automated, high-throughput conversion of skin fibroblasts into iPSCs and their characterization/differentiation with minimal manual intervention [157]. However, it still remains unknown how to obtain qualified iPSCs and improve the quality of patient-specific iPSCs under suitable and cost-effective cultivation conditions for diverse applications, including disease modeling, drug screening, and customized therapy. As mentioned above, although iPSCs are similar to ESCs in terms of pluripotent marker gene expression, self-renewal potency, differentiation potential, and their morphology, they are not identical. Recent extensive genetic analysis using high-throughput sequencing technology or generation of single-nucleotide genome-wide maps of DNA methylation has demonstrated the genomic/epigenetic differences between iPSCs and ESCs. However, the functional consequences of their differences *in vitro* or *in vivo* are largely unknown. Moreover, iPSCs and ESCs show a wide range of clonal variations in terms of proliferation and differentiation potential. Furthermore, iPSCs derived from even the same parental somatic cells have different potential in terms of expansion or differentiation [158–160]. Considerable somatic coding mutations occurring in hiPSC lines have also been reported by extensive exome analysis [161]. The other main concern is the instability of iPSCs during passaging of clones. It has been reported that early passages of iPSCs display *de novo* copy number variations (CNV) during the reprogramming process [162]. Thus, to obtain a more reliable outcome from iPSC research, generation of isogenic iPSCs using recent gene engineering technology or by establishment of at least 2–3 iPSC clones from the same parental somatic cells has been suggested. However, it remains unknown how these genetic/epigenetic alterations occur during reprogramming or expansion of iPSCs as well as how these alterations can be managed for iPSC generation or its application. Further, it remains unknown whether there is any reprogramming method to reduce or exclude these possible alterations as well as how to select the qualified iPSC clone from a variety of iPSC lines. To address these questions, further intensive works at the genetic/epigenetic/cellular levels are needed, and *in vivo* functional characterization of iPSC-derived cells needs to be carried out. Thus, the most important issue in iPSC generation is to establish more stable and standard protocols for safer and easier iPSC generation in diverse applications.

Although there are some differences between ESC and iPSCs, iPSCs are still the most promising choice for modeling with human cells. In mouse, iPSCs have the same potential as ESCs because a mature organism can be generated from iPSCs via blastocyst injection or tetraploid complementation [163]. Although human iPSCs cannot be tested using these embryological methods owing to ethical issues and hiPSCs appear to be ‘primed’ PSCs as mouse Epi-stem cells, naïve human PSCs might be used as another human cellular model.

#### Current limitations of studies on pathophysiology using ASD iPSC-derived neurons

Besides iPSC line-to-line variations, limitations of studying ASD with hiPSC-derived neurons include phenotypic variations between neurons derived from the same iPSCs, which are based on differences between individual hiPSC-derived neurons from even a single patient due to heterogeneity of neuronal subtypes differentiated from each iPSC line [164] even with well-defined differentiation protocols. In addition, different differentiation methods such as usage of small molecules or genes, EB formation vs. monolayer culture, concentration of small molecules and growth factors, differentiation time can also generate variations in the neuronal population. The use of cell type-specific promoters to drive expression of fluorescent markers for purification by cell sorting or identification of desired cell types would be a powerful tool to reduce variation. The surrounding environment of cells may also significantly affect the phenotypes. For example, the presence of neural progenitor cells in neuronal culture could mask disease-associated phenotypes by continuous production of newborn neurons [112]. Therefore, to obtain reliable data using hiPSC-derived neurons from ASD patients, each experiment should be performed with multiple neuronal differentiation protocols from at least two or three independent hiPSC lines with the same mutation from multiple patients. In addition, forced expression of a transcription factor like *Ngn2* would be a good method to overcome the above described issues, in which almost ~100 % of cortical neurons at a similar maturation stage could be generated, and the neurons showed their synaptic phenotypes as early as 3 weeks after forced Ngn2 expression [97].

As mentioned above, widespread genetic variations could exist between iPSC lines themselves derived from unrelated individuals. Therefore, genetically related family member-derived control lines could possibly be used to reduce variability of phenotypes, although it would not completely remove the possibility that even a single genetic difference could potentially affect observable phenotypes. Another possible way is to use gene correction methods since the ideal controls would be those that have the same genetic background except only the specific genetic defect found in the patient. Many well-known syndromic ASD-related genetic variants can be modeled with “isogenic” cell lines, where a patient-derived iPSC line could be gene-corrected using ZFNs, TALENs, or CRISPR-Cas9 technologies, reverting a mutant line to wild-type or vice versa [165, 166].

As iPSCs are an *in vitro* culture system, they lack many characteristics of a developing and mature brain physiology *in vivo*. Therefore, it is difficult to study neuronal circuitry and organization using iPSC-derived neurons under 2D conditions, particularly when investigating phenotypes unique to specific neuronal circuits of the adult brain. One way to avoid these problems is to xenograft iPSC-derived neural progenitor cells (NPCs) into embryonic rodent brains to allow them to integrate into developing neural networks and mature *in vivo*. Cerebral “organoids” [130] is another possible way to study disease phenotypes in a specific cell type or group of cell fates in the context of 3D model of human neurodevelopment [130, 167], as reported by Mariani et al. [132].

In summary, we can generate hiPSC-derived neurons from fibroblasts and other somatic cells of ASD patients to investigate alterations of neuronal connectivity, synaptic maturation, and functions. In addition, direct conversion of fibroblasts from ASD patients into neurons or NPC/NSCs would be used as an alternative *in vitro* model of ASD in the near future. However, we need to realize that hiPSC-based studies of ASD pathophysiology will not completely replace human postmortem and mouse genetic studies. Nevertheless, disease modeling with hiPSC-derived neurons combined with their comprehensive molecular and functional characterization will be a new and strong tool for understanding complex neurodevelopmental disorder, ASD.

## Abbreviations

ASD: Autism spectrum disorder
iPSC: induced pluripotent stem cell
ESCs: Embryonic stem cells
PSCs: Pluripotent stem cells
ZFN: Zinc finger nuclease
TALEN: Transcription activator-like effector nuclease
HAC: Human artificial chromosome
Wnt: Wingless/Int proteins
BMP: Bone morphogenic protein
SHH: Sonic hedgehog
FGF8: Fibroblast growth factor 8
RA: Retinoic acid
EB: Embryoid body
bFGF: basic fibroblast growth factor
TGFβ: Transforming growth factor β
*Ngn2*: *Neurogenin 2*
MRI: magnetic resonance imaging
2D: Two-dimensional
3D: Three-dimensional
RTT: Rett syndrome
*MECP2*: Methyl-CpG binding protein 2
TS: Timothy syndrome
TH: Tyrosine hydroxylase
TRPC6: Transient receptor potential channel 6
CNV: Copy number variation
